# Comparative Toxicological In Vitro and In Ovo Screening of Different Orthodontic Implants Currently Used in Dentistry

**DOI:** 10.3390/ma13245690

**Published:** 2020-12-13

**Authors:** Camelia A. Szuhanek, Claudia G. Watz, Ștefana Avram, Elena-Alina Moacă, Ciprian V. Mihali, Adelina Popa, Andrada A. Campan, Mirela Nicolov, Cristina A. Dehelean

**Affiliations:** 1 Department of Orthodontics, Faculty of Dental Medicine, Victor Babeş University of Medicine and Pharmacy, 9 No., Revolutiei Bv., 300041 Timişoara, Romania; cameliaszuhanek@umft.ro (C.A.S.); popa.adelina@umft.ro (A.P.); 2 Departament of Pharmaceutical Physics and Biophysics, Faculty of Pharmacy, Victor Babeş University of Medicine and Pharmacy, 2nd Eftimie Murgu Sq., 300041 Timişoara, Romania; nicolovmirela@gmail.com; 3 Department of Pharmacognosy, Faculty of Pharmacy, Victor Babeş University of Medicine and Pharmacy, 2nd Eftimie Murgu Sq., 300041 Timişoara, Romania; stefana.avram@umft.ro; 4 Departament of Toxicology, Faculty of Pharmacy, Victor Babeş University of Medicine and Pharmacy, 2nd Eftimie Murgu Sq., 300041 Timişoara, Romania; alina.moaca@umft.ro (E.-A.M.); campan.andrada@yahoo.com (A.A.C.); cadehelean@umft.ro (C.A.D.); 5 Department of Life Sciences, Faculty of Medicine, Vasile Goldis Western University of Arad, 86 No., Liviu Rebreanu St., 310414 Arad, Romania; mihaliciprian@yahoo.com; 6 Molecular Research Department, Research and Development Station for Bovine, 32 No., Bodrogului St., 310059 Arad, Romania

**Keywords:** orthodontic implants, surface topography, cytotoxicity, HGF, HET-CAM assay

## Abstract

Selecting the most biocompatible orthodontic implant available on the market may be a major challenge, given the wide array of orthodontic devices currently available on the market. The latest scientific data have suggested that in vitro evaluations using oral cell lines provide reliable data regarding the toxicity of residual particles released by different types of orthodontic devices. In this regard, the in vitro biocompatibility of three different commercially available implants (stainless steel and titanium-based implants) was assessed. Methods: As an in vitro model, human gingival fibroblasts (HGFs) were employed to evaluate the cellular morphology, cell viability, and cytotoxicity by means of 3-(4,5-dimethylthiazol-2-yl)-2,5-diphenyltetrazolium bromide (MTT) and lactate dehydrogenase (LDH) assays at 24 h and 72 h post-exposure to test implants. Results: The results correlate the composition and topography of the implant surface with biological experimental evaluations related to directly affected cells (gingival fibroblasts) and toxicological results on blood vessels (hen’s egg test-chorioallantoic membrane (HET-CAM) assay). The stainless steel implant exhibits a relative cytotoxicity against HGF cells, while the other two samples induced no significant alterations of HGF cells. Conclusion: Among the three test orthodontic implants, the stainless steel implant induced slight cytotoxic effects, thus increased vigilance is required in their clinical use, especially in patients with high sensitivity to nickel.

## 1. Introduction

Bone screws, known as temporary anchorage devices (TADs), were first introduced to orthodontics in 1983 by Creekmore and Eklund for treatment of the deep bite. Since then, they have been used for other orthodontic movements, such as space closure, correction of asymmetric cant, or en-masse retraction [1]. However, another reason orthodontic implants are widely used is related to the aesthetic aspect, in which people are becoming more and more interested [2].

Orthodontic implants are small devices that are implanted by an easy surgery and increase the success rate of orthodontic treatment [3]. Although they have relatively large proportions that limit their functionality, they also have some advantages, such as visibly decreased anatomic limitations, minimally invasive surgery, and reduced cost—aspects that increase patients’ compliance [4].

Orthodontic micro-implants are temporary anchorage devices (TADs) used for orthodontic treatment that possess a success rate of 80% [5]; however, the reliability and survival rate of orthodontic implants are directly influenced by primary stability, considered to be the most important parameter. Unfortunately, the factors that influence primary stability are not yet elucidated, but are related to implant design and the surgical procedure employed. The method that evaluates the quality of primary stability is called measurement of insertion torques. Nevertheless, almost as important as primary stability is bone quality and oral hygiene [6,7,8].

Another factor that plays an important role in the success of orthodontic implants is the material from which the implants are manufactured. The raw material must meet certain characteristics, such as non-corrosivity, biocompatibility, non-toxicity, and mechanical resistance, to be considered suitable for orthodontics [1]. A material that simultaneously fulfills all the above-mentioned features is titanium, thus it is the most commonly used material for the manufacture of TADs [1,9,10]. However, for better resistance, multi-metal alloys such as aluminum, vanadium, or nickel are preferred [10,11]. Nevertheless, it is also important that the internal structure of the implant is continuous and homogeneous [10]; moreover, the higher the roughness of the implant material, the greater the clinical success of the orthodontic implant as a result of improved bone–implant contact [12]. Moreover, the chemical composition of the metal alloy offers specific physico-chemical features to orthodontic materials that are of significant importance for the biomechanics of the tooth movement [13].

Multiple studies have investigated the release of metal ions from orthodontic implants in saliva or blood [1,14,15,16], revealing that, although there is an amount of ions’ release, the concentration is not considered toxic. Nevertheless, this cannot guarantee that the cells in the buccal mucosa have not undergone morphological changes [1,14]. Still, even if the amount of ions released by implants was considered non-inflammatory for the body [15], oral hygiene and micro-implant head shape were associated with the inflammation process [5]. However, ions’ release can activate the immune cell line and may also induce mutagenic or carcinogen effects known not to be necessarily dose-dependent [14,15]. Although carcinogenic effects cannot be predicted, it is particularly important to know if metal ions released from orthodontic implants lead to cellular toxicity [15], thus basic cytotoxic investigations could be employed to evaluate the effect of ions released from orthodontic materials on buccal mucosa cells [1].

It is also important to mention that the success rate of orthodontic implants is based on several key factors, such as the implantation procedure employed, dental material features, implant topography, and design [17,18].

In regard to the aspects presented above, the present study aims to reveal the biosafety profile of three different orthodontic implants by correlating the surface topography of each implant with the biological results obtained following their exposure to gingival fibroblasts, starting from the premise that the composition of the metal alloy together with the surface topography of the implant play an important role in ensuring the biocompatibility of the orthodontic devices. Thus, the biosecurity profile of one stainless steel orthodontic implant (Leone, Italy) and two titanium-based implants from Abso Anchor Dentos (Daegu, Korea) and psm Medical Solution Gmbh (Gunningen, Germany) was assessed through the extraction means method in terms of cell morphological aspects, cell viability, and cytotoxicity by employing an in vitro model represented by primary human gingival fibroblasts (HGFs) cells. Moreover, the potential irritative effect of the release of the metal ions from orthodontic implants has also been investigated in vivo, using the hen’s egg test-chorioallantoic membrane (HET-CAM) assay. The technique is an alternative method to animal testing used for the evaluation of biocompatibility and potential irritative effects on skin or mucosal tissues table [19] and was previously applied for some types of dental materials [20,21]. It involves the exposure of investigated extractive solutions of the orthodontic implants to the developing blood vessels of the CAM, monitoring any signs of disruptive events.

## 2. Materials and Methods 

**Figure 1:**
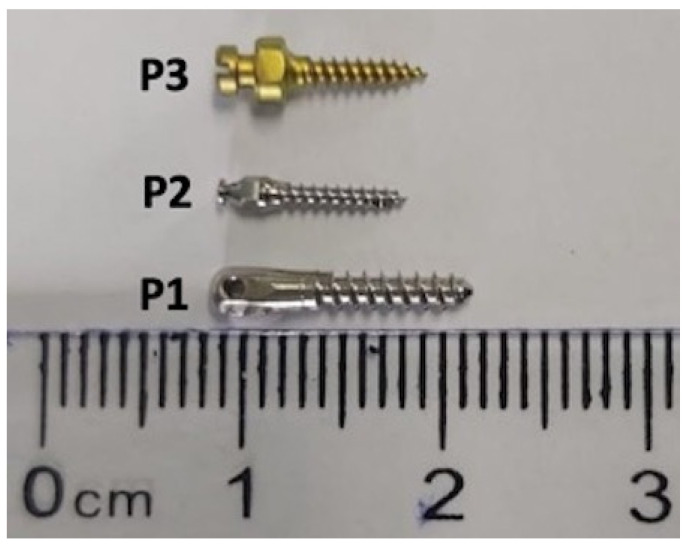
Representative image of test orthodontic implants.

In the current study, three different types of orthodontic implants were used, namely, P1 = Stainless Steel Orthodontic Mini-implant, (Leone, Italy); P2 = Orthodontic Micro-implant Abso Anchor, Dentos (Daegu, Korea)—a titanium-based alloy (Ti6Al4Va) [22]; and P3 = psm Titanium Implant, psm Medical Solutions Gmbh (Gunningen, Germany) (Figure 1).

Because of the very large and complex surface of the implants, they cannot be tested in vitro by direct exposure to cell cultures, thereby the extraction method was employed. For this method, all test orthodontic implants were maintained in cell culture medium for 24 h, at 37 °C, and intermittent shaking to simulate the physiological conditions of the oral cavity [1]. This technique offers the possibility to evaluate the in vitro cytotoxicity of medical devices by extraction means, according to International Standard Organization (ISO standard 10993-5:2009) [23]. The same extraction medium was used for the in vivo evaluation of the irritation potential in the HET-CAM assay.

In brief, the cell culture medium that included the residual ions/substances released by test orthodontic implants was further used to stimulate the primary human gingival fibroblasts (HGFs) and the chorioallantoic membrane.

### 2.1. SEM Assessment of Orthodontic Implants Surface

In order to investigate the qualitative surface topography of the test orthodontic implants, scanning electron microscopy (SEM) was employed. SEM was performed using a FEI Quanta 250 microscope. Pictures were taken at two magnification orders—for a general overview and for higher surface topography side’s analysis, respectively. Macro-scale differences were assessed at 50× power magnification and 3 mm working distance. Higher magnification (3k×) was used to evidence the micro topographies.

### 2.2. Cell Line and Culture Conditions

The cells used in the present study were primary human gingival fibroblasts (HGFs) cells acquired from American Type Culture Collection (ATCC, code no PCS-201-018™). The complete cell growth medium was prepared from Fibroblast Basal Medium (ATCC® PCS-201-030™) supplemented with Fibroblast Growth Kit-Low serum (ATCC® PCS-201-041™) and 0.1% Penicillin-Streptomycin-Amphotericin B Solution (ATCC® PCS-999-002™). All experiments were performed under standard conditions; the cells were grown in a humidified atmosphere with 5% CO_2_ and 37 °C in a Steri-Cycle i160 incubator (Thermo Fisher Scientific, Inc., Waltham, MA, USA) and all of the methods were performed under sterile conditions within a biosafety class II cabinet (MSC Advantage 12 model (Thermo Fisher Scientific, Inc., Waltham, MA, USA).

### 2.3. Cell Morphology Assessment

After exposure to the extraction medium of test orthodontic implants, the morphology of HGF cells was evaluated by taking pictures at magnification of 20× in early stages (3 h) and consistent times (24 and 72 h), using an Olympus IX73 inverted microscope equipped with DP74 camera (Olympus, Tokyo, Japan).

### 2.4. MTT (3-(4,5-dimethylthiazol-2-yl)-2,5-diphenyltetrazolium bromide) Assay

The viability of HGF cells treated with the extraction medium of test orthodontic implants was evaluated by the means of MTT technique. This method assesses the activity of mitochondrial dehydrogenase from metabolically viable cells. The effect can be quantified through a colorimetric reaction in which the MTT (yellow compound) is reduced by viable cells to formazan (dark blue compound). The protocol was performed as previously described in one of our studies [24]. Briefly, HGF cells to a density of 10^4^ cells/well were seeded in 96-well plates and incubated overnight. The next day, the cells were treated with the extraction medium for 24 h. After that, the medium was replaced with a fresh one, followed by the addition of 10 μL MTT reagent and the plate was incubated for 3 h. The formazan crystals formed in these 3 h were solubilized with 100 μL solubilization buffer/well, followed by 30 min incubation at room temperature. The absorbance of each well was measured spectrophotometrically at 570 nm wavelength with a microplate reader (xMark™ Microplate; Bio-Rad Laboratories, Inc., Hercules, CA, USA).

### 2.5. Lactate Dehydrogenase (LDH) Assay

LDH assay was employed to evaluate the cytotoxic effect induced by extraction medium of test micro-implants (P1, P2, P3) on primary human gingival fibroblasts (HGFs) cells. The principle of this technique is based on the quantification of the cytosolic enzyme LDH that could be released into the extracellular medium when cellular membrane damage occurs. The protocol performed for LDH analysis was similar with the one described for MTT assay, with some exceptions [25,26]. On the day of the assay, 50 μL per well of stimulation medium was transferred into a new 96-well plate and mixed with 50 μL per well of reaction mixture. After that, the plate was incubated for 30 min at room temperature and, finally, the reaction was stopped by adding 50 μL/well of stop solution. The LDH amount released into the medium was determined spectrophotometrically by reading the absorbances of each well at the wavelengths of 490 nm and 680 nm with a microplate reader (xMarkTM Microplate; Bio-Rad Laboratories, Inc., Hercules, CA, USA).

### 2.6. HET-CAM Assay

In order to detect a possible irritative reaction on gingival tissues, the orthodontic implants extraction medium was assessed using the HET-CAM technique, according to Interagency Coordinating Committee on the Validation of Alternative Methods (ICCVAM) guidelines (Interagency Coordinating Committee on the Validation of Alternative Methods [27]).

Briefly, following a slightly modified basic protocol, fertilized eggs from Gallus gallus domesticus were disinfected and placed in the incubator in a horizontal position, under constant conditions of temperature (37 °C) and humidity. On the third day of incubation, a volume of 5–7 mL of albumen was extracted to allow the chorioallantoic membrane to detach from the eggshell so that the blood vessels could be more easily observed. On the fourth day of incubation, a window was cut in the upper part of the egg, which was then covered with adhesive tape, and the eggs were reintroduced into the incubator until the day the experiment began [28,29].

Samples were applied in a volume of 200 µL, and changes in blood vessels were observed using a stereomicroscope (Discovery 8 Stereomicroscope, Zeiss, Göttingen, Germany). The images were taken with Axio CAM 105 color, Zeiss, before and five minutes after applying the solution. Finally, the images were processed using the ImageJ v 1.50e program (U.S. National Institutes of Health, Bethesda, MD, USA).

PBS (phosphate buffered saline) was used for the negative control, and a solution of 0.5% sodium lauryl sulfate (SLS) was used as a positive control. After application for five minutes (300 s), the following effects were observed in the blood vessels: hemorrhage (*H*), lysis of the vessels (*L*), and coagulation (*C*). The potential irritant effect was expressed by calculating the irritation score (*IS*) using the following equation:

(1) $$IS=5\times \frac{301-H}{300}+7\times \frac{301-L}{300}+9\times \frac{301-C}{300}$$

Using a scale introduced by Luepke, the irritating effect was expressed as an irritation score (*IS*) ranging from 0 to 21, which allows the classification of the effects into four categories ranging from non-irritant to strong irritant [30].

### 2.7. Statistical Analysis

The Graph Pad Prism 5.0 Software (San Diego, CA, USA) was employed for data presentation and statistical analysis. The results were obtained from three independent experiments and are presented as the mean value ± standard deviation (SD). One-way analysis of variance (ANOVA) followed by Tukey’s post-test was performed to evaluate the statistically significant differences between experimental and control groups; *** indicates *p* < 0.001.

## 3. Results

### 3.1. SEM Analysis 

**Figure 2:**
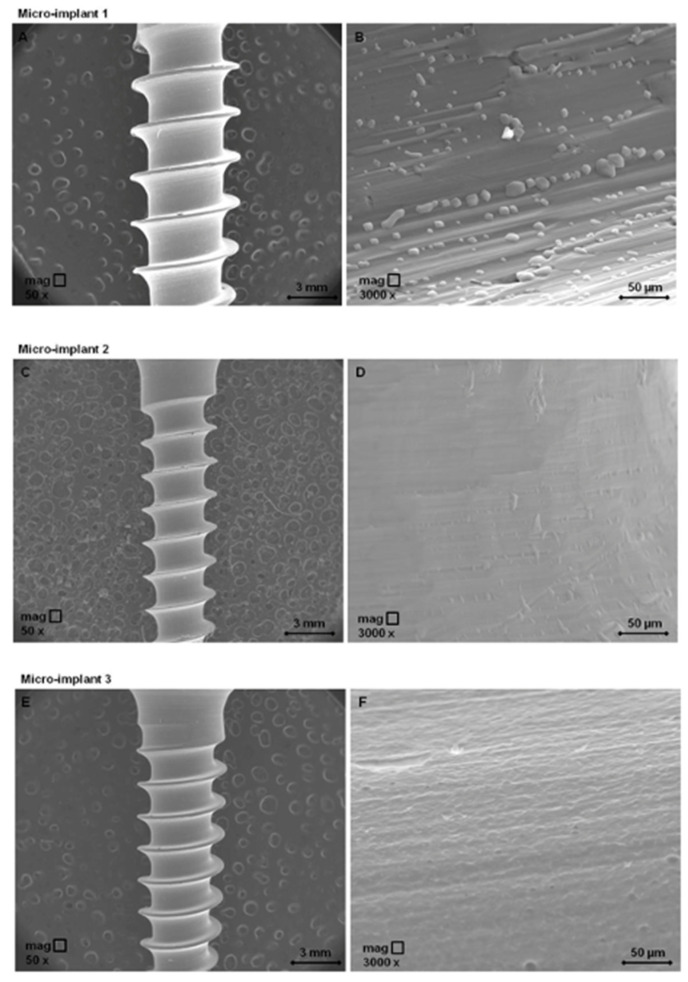
Scanning electron microscopy micrographs of test orthodontic implants at two different magnification orders. Pictures (**A**,**C**,**E**) show the general overview of the test orthodontic implants—P1, P2, P3, respectively by employing an enlargement of 50×; Pictures (**B**,**D**,**F**) show the surfaces’ detail topography of the test orthodontic implants P1, P2, P3, respectively by employing 3000× magnification.

As presented in Figure 2, SEM micrographs revealed that the P1 sample exhibits a roughened topography micro-structure with an indefinite shape like grain structure type, while SEM micrographs of the P2 sample showed a smooth surface with slight elongated unevenness, from place to place. The P3 sample possesses a microstructure with a porous surface, with elevations and depressions such as rounded holes.

### 3.2. Cell Morphology

**Figure 3:**
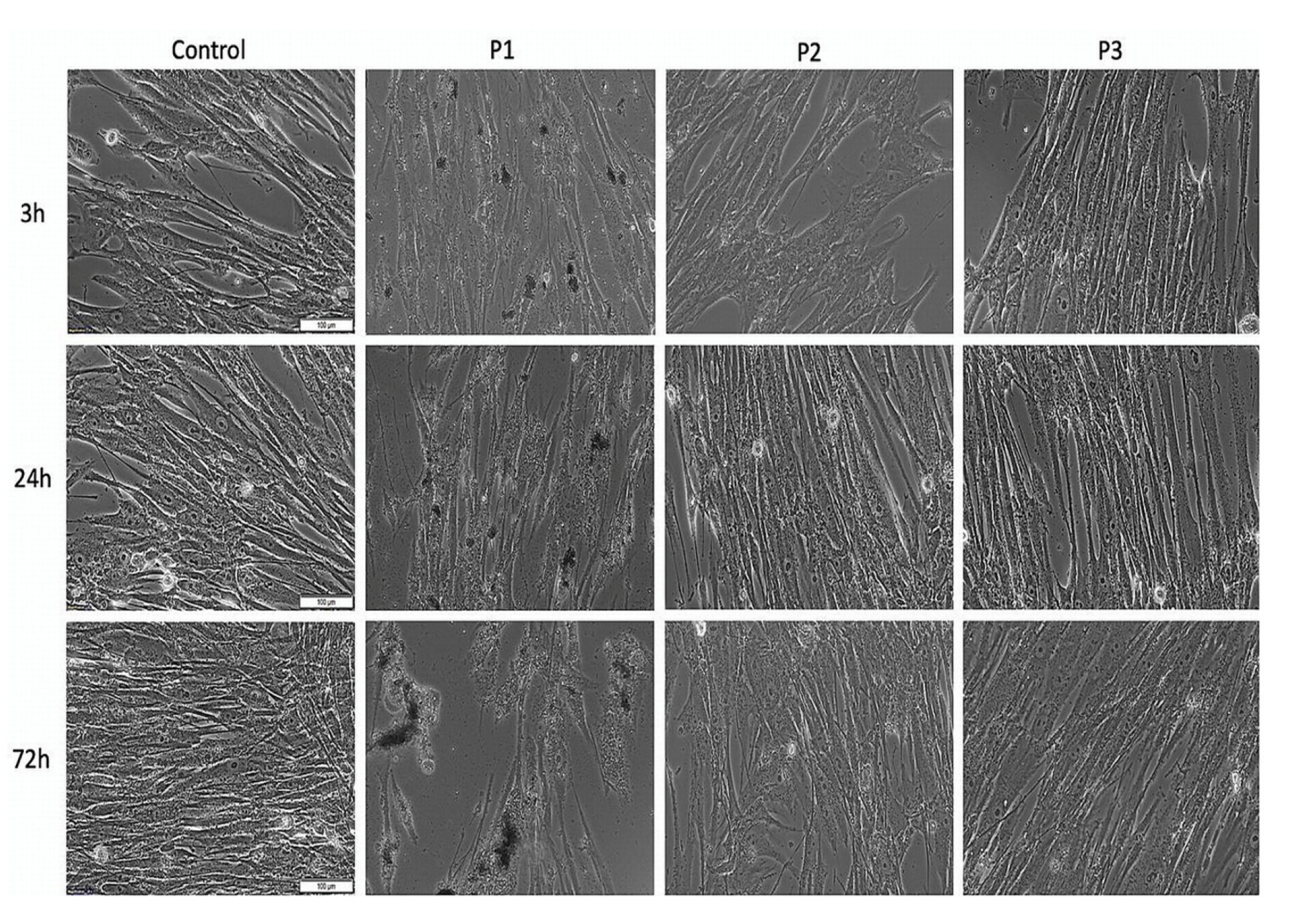
Morphological aspects of primary human gingival fibroblasts (HGFs) cells after treatment with extraction medium of test orthodontic implants (P1, P2, P3). Pictures were captured at a magnification of 20×. Scale bars represent 100 μm.

As shown in Figure 3, the control HGF cells treated with specific growth medium presented an elongated spindle-like shape and manifested high confluence. However, HGF cells treated with the extraction medium corresponding to P1 seemed to be stressed (low intensity) after 3 h post-treatment. In addition, the effect was augmented by increasing the incubation time, with the HGF cells displaying some cell morphological alteration and cell density decrement, following a stimulation of 72 h. Still, the HGF cells treated with the extraction medium of P2 and P3 samples did not show significant changes of cell morphology compared with control cells after a stimulation of 24 h. However, at 72 h post-treatment, HGF cells exposed to P2 extraction medium exhibited some morphological changes—several cells did not present the specific spindle-like shape, but not very relevant as an experimental screening. When referring to HGF cells exposed to P3 extraction medium, there was no significant morphological alteration observed even after 72 h stimulation time.

### 3.3. Cell Viability Assay by Means of MTT Analysis

**Figure 4:**
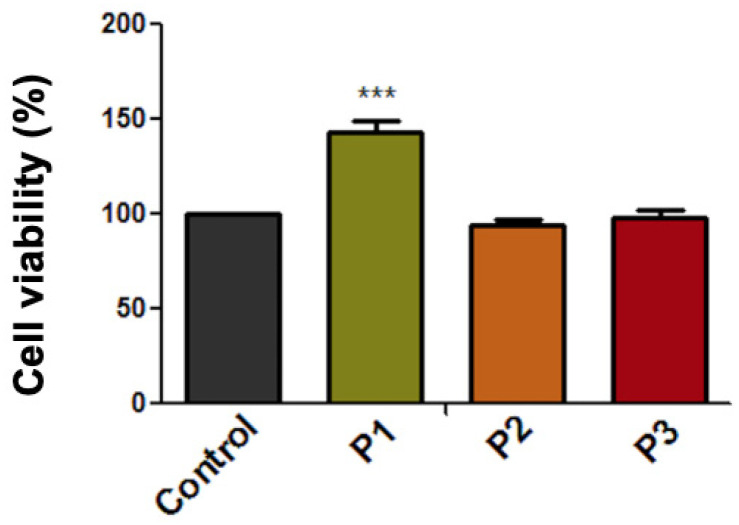
Cell viability percentage of primary human gingival fibroblasts (HGFs) cells at 24 h post-treatment with extraction medium of test orthodontic implants (P1, P2, P3). Data are presented as cell viability percentage (%) normalized to control cells. The results represent the mean values ± SD of three separate experiments. One-way analysis of variance (ANOVA) test was performed to determine the statistical differences followed by Tukey’s multiple comparisons analysis (*** *p* < 0.001).

As presented in Figure 4, the viability rate of HGF cells after exposure to the extraction medium of test samples (P1, P2, P3) was above 90%. Moreover, the cell viability rate of the cells treated with the P1 sample was above 143%, showing a proliferative effect. However, this result was considered a false positive effect and could be caused by the metallic particles released into the extraction medium and their further interaction with the MTT reagent. This type of interaction has been previously identified in several in vitro studies [31,32,33]. Still, the P2 and P3 samples did not interfere with the MTT technique, as their corresponding micro-implants did not release metallic microparticles in the extraction medium, as could be easily observed from Figure 3. Thus, the cell viability rates of P2- and P3-treated cells are considered relevant and are as follows: 93.83% and 98.37%, respectively.

Because of the limitations presented by the MTT method, the LDH assay was also employed to assess the cytotoxic effect of the test samples, at two different time intervals.

### 3.4. Evaluation of the Cytotoxic Effect through LDH Release Method

**Figure 5:**
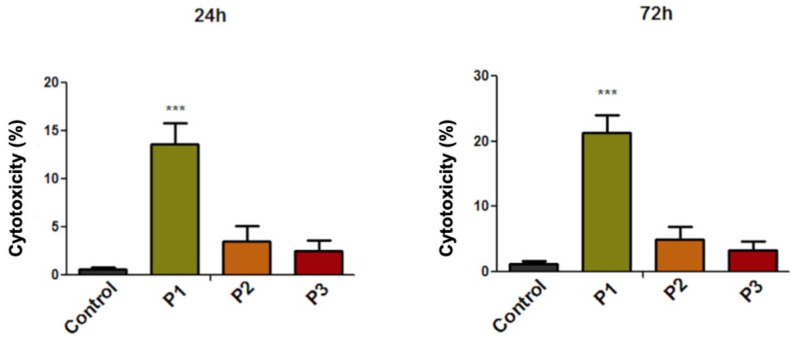
Cytotoxicity percentage of primary human gingival fibroblasts (HGFs) cells at 24 h and 72 h post-treatment with extraction medium of test orthodontic implants (P1, P2, P3). The results represent the mean values ± SD of three separate experiments. One-way ANOVA test was performed to determine the statistical differences followed by Tukey’s multiple comparisons analysis (*** *p* < 0.001).

The lactate dehydrogenase (LDH) assay revealed that only the P1 sample induced a slight cytotoxic effect on the HGF cell population, at 24 h and 72 h post-treatment. As expected, the cytotoxic effect increased with the stimulation time, with the HGF cells displaying a cytotoxic rate of 13.64% after treatment with the P1 sample at 24 h post-incubation and a cytotoxic effect of 21.39% when the HGF cells were treated with P1 sample for a period of 72 h. The cytotoxic effects induced by samples P2 and P3 were not significant, with the HGF cells developing a cytotoxic rate under 5% (Figure 5).

### 3.5. Irritation Assessment Using the HET-CAM Test

**Figure 6:**
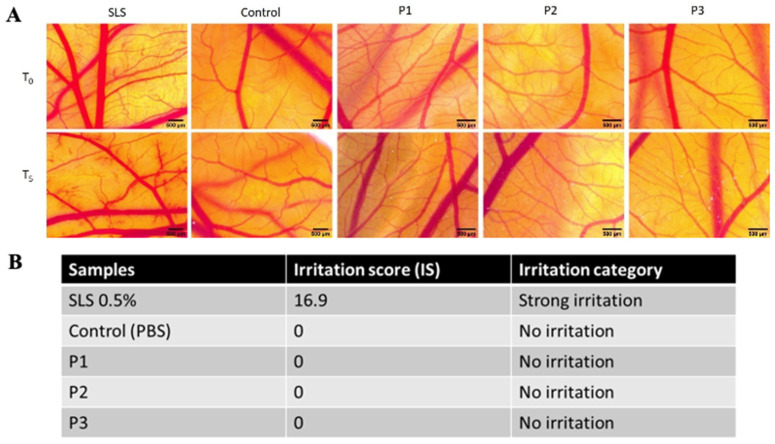
Irritation evaluation using the hen’s egg test-chorioallantoic membrane (HET-CAM) assay. (**A**) Stereomicroscopic images of the CAM vasculature before treatment (T0) and 5 min after treatment with extraction medium of test orthodontic implants P1, P2, and P3, next to sodium lauryl sulfate (SLS) 0.5% (positive control) and phosphate buffered saline (PBS) (negative control); scale bars 500 µm. (**B**) Irritation scores of the test orthodontic implants P1, P2, and P3. The experiment was performed in triplicate.

By evaluating the extraction medium of the implants, none of the three tested samples induced any considerable alteration upon the monitored parameters. During the 5 min of observation, no sign of hemorrhage, coagulation, or vessel lysis was registered for the three test samples as well as for the control. In contrast, severe events were recorded as being induced by the positive control, SLS, as observed in CAM features when exposed to treatment, and expressed by the irritation scores and the irritation categories (Figure 6A,B). After a short-term exposure (5 min) to the extraction medium of the implants, all three samples showed no irritation potential, thus indicating a good biocompatibility potential with vascular mucosal tissues.

## 4. Discussion

Biocompatibility is one of the most important features of orthodontic materials in terms of clinical use [1]. However, scientific data reveal that physical and chemical properties of the orthodontic devices such as bands, brackets, mini-screws, and archwires influence the biosafety of the orthodontic materials, especially owing to metal ions’ release [13]. These results are related to both in vivo and in vitro studies performed under specific experimental conditions and of particular interest owing to possible mutagenic, cytotoxic, and immunogenic effects that may be caused by the orthodontic devices [13,14,15].

In vitro oral models are often used to evaluate the biocompatibility of dental materials, as they present several advantages when compared with in vivo experiments, such as the ease of reproducibility, cost saving, and performance under well-controllable parameters [16]. In addition, in vitro assays offer reliable data concerning the proliferation and cellular events of different oral cell lines exposed to a wide variety of bio-chemical compositions of the dental materials [34]. Nevertheless, in vitro experiments must respect ISO standard 10993-5:2009 [23]. Regarding this aspect, the present study was conceived in accordance with ISO regulations, performing all in vitro experiments by extraction means—maintaining the test mini-implants in complete cell culture media for 24 h. Thereafter, the collected medium was applied on the top of the cells for different time intervals—24 h and 72 h, respectively. The in vitro model employed in the present study consists of primary human gingival fibroblasts (HGFs), because, under normal physiological conditions, these cells are directly exposed to the released ions from orthodontic implants [35] and, compared with osteoblasts, HGF cells are rarely subjected to in vitro studies that assess dental implant biocompatibility/cytotoxicity. Beside this, gingival fibroblasts play a key role in the generation of the soft tissue surrounding the implant and are also involved in the generation of specific pro-inflammatory markers, such as IL-6, IL-8, or monocyte chemoattractant protein-1 (MCP-1). However, the inflammatory process is usually experienced when epithelial disruption occurs and is generated as a response to different bacterial moieties from the oral cavity [9]. Nevertheless, in a recent study, Andrukhov and collaborators [9] discussed the implications of gingival fibroblasts in initiating the inflammatory process following exposure to two types of materials (titanium and zirconium) that are commonly used to obtain dental implants. Thus, it was revealed that the production of IL-6 and MCP-1 is correlated with the surface proprieties (topography and roughness) of the implant material, whereas IL-8 production was barely impaired.

Similar aspects related to the influence of orthodontic implant surface on implant cytocompatibility were also observed in the present study. Thus, the slightly grooved surface of the stainless steel implant possesses several polygonal shapes (Figure 2B), which may be a source of possible contaminating particles. Their presence and persistence on the sample surface (compared with the samples P2 and P3) may indicate a certain affinity of the material for adhesion, but also for inorganic and organic contaminants when it comes into contact with periodontal tissue, which may induce pro-inflammatory reactions by releasing specific cytokines and chemokines [36].

Regarding the biological profile of the stainless steel orthodontic implant, under the current experimental conditions, the results revealed that P1 samples induced some morphological alteration of HGF cells, especially at 72 h post-exposure (Figure 3). These data were further supported by the LDH release analysis, which revealed a relative cytotoxic percentage of 13.64% and 21.39%, after a stimulation time of 24 h and 72 h, respectively. However, even if the LDH assay indicated a relative cytotoxicity, according to ISO Standard 10993-5:2009 regarding Biological Evaluation of Medical Devices [23], a sample is considered cytotoxic if the viability of the cells is reduced by more than 30%, thus, based on this consideration, the implant could not be considered cytotoxic.

Nevertheless, because the structural composition of the stainless steel orthodontic mini-implant (Leone, Italy) is mainly based on iron, nickel, and chromium [35], additional caution should be taken when this type of implant is intended to be used in patients who present a relative sensitivity to Ni. As this element is considered to be the main metal responsible for inducing allergies, especially in women (up to 20%), which is more likely to be caused by repeated exposure to jewelry that contains Ni [37,38]. Thereby, the source of Ni provided by orthodontic devices should not be ignored, as Ni is employed in a great array of metal alloys used in dentistry and limited data are available regarding Ni discharge from orthodontic devices [39], which could be an important trigger for allergies, as severe allergenic reactions have already been reported in a case report study of a young woman with allergy-free medical history who presented Class I malocclusion [40].

Nevertheless, besides Ni, two other metals that are commonly used in obtaining dental metal alloys are associated with cytotoxic reactions, including chromium (Cr) and cobalt (Co) [41], both of which have already been reported to induce inflammatory reaction in HGF cells and osteoblasts by modulating several signaling pathways, such as NF-kB and MAPKs [42], while Ni and Cr concentrations up to 0.78 ng/mL were identified in buccal mucosa cells of patients who have had orthodontic appliances for a period of six months, inducing important cell viability decrease and DNA impairment, which were observed mainly after the first three months [43].

Micro-implant P2 surface (Figure 2D) shows longitudinal surface depressions with micro unevenness at the surface, with the surface exhibiting a generally smooth appearance compared with sample P3. Meanwhile, a micro-grooved surface observed in micro-implant sample P3 (Figure 2F), may represent the proper surface structure that could be placed in contact with periodontal ligament tissue and, consequently, to determine a periodontal ligament formation. Nevertheless, it is also important to evaluate the behavior of human gingival fibroblast to titanium implants from the perspective of their bacterial potential, as it is well known that titanium/titanium alloys are prone to bacterial colonization leading to inflammation [44]. He and co-workers [45] stated that bacterial colonization is more decreased on zirconia implants compared with Ti-based dental implants, while Liu’ group attested the anti-bacterial effect of copper-bearing titanium alloy after exposure to Staphylococcus aureus and Escherichia coli, indicating antibacterial rates of 99% after 24 h [44].

Compared with P1, the stainless steel implant, P2 and P3 samples titanium-based devices could be considered superior orthodontic implants in terms of biocompatibility, as they (i) did not manifest morphologic alteration on HGF cells; (ii) did not reduce the cell viability of the HGF cells; and (iii) did not induce cytotoxic events of the HGF cell population. However, compared with our results, Malkoc S et al. [35] obtained contradictory results when different mini-implants obtained from stainless steel and titanium alloy were screened for a possible cytotoxic effect on gingival fibroblasts. Their study revealed that none of the test orthodontic implants induce a cytotoxic potential on gingival fibroblasts. Nevertheless, the same study sustained that Leone orthodontic mini-implant (the same implant noted as P1 in this study) manufactured from 316 stainless steel induced a significant inhibitory potential on murine osteoblasts (MC3T3) E1 cells [35]. The contradictory results reported by Malkoc’s group on gingival fibroblasts could be caused by the filtration step of the culture medium before it was applied on the top of the culture cells, as this procedure could have removed some residual particles released by the orthodontic implant, consequently leading to a reduction of its cytotoxic potential. However, there are several data regarding the toxicity of titanium (Ti) particles depending on their dimension, when employing in vitro assessments [46], with several tests on fibroblasts and osteoblasts being applied for this aim; showing that nanosized Ti particles accumulated in a higher proportion within periodontal ligament fibroblasts compared with micro-sized Ti particles; however, the inflammatory effects seem to not be notable [46].

Regarding the in vivo assessment, by exposing the chorioallantoic membranes to the extraction medium of the implants for longer interval, some differences were observed among the three tested samples. In terms of viability, P1 induced a shorter post-treatment survival rate (5 days), as compared with P2 and P3 (7 days). This long-term investigation outcome can be correlated with the higher cytotoxic effect induced by P1 and possibly explained by the metallic particles released into the extraction medium and their influence upon the developing CAM. As observed by others [20], certain metal elements present in an extraction medium might influence their cytotoxicity, but not necessarily represent an irritant material when evaluated in an in vivo HET-CAM setting.

Summarizing the results obtained in the present study, there are no significant concerns raised by titanium-based implants for their further clinical use in orthodontics, as Ti is considered to be the most biocompatible metal, especially because of its high corrosion resistance compared with other metals used in orthodontics and also because of its proper integration with the bone surrounding tissue [47].

Nevertheless, the limitation of the present study derives from the fact that the orthodontic implants were maintained for a short period of time in the cell medium and have not been exposed to variations of physiological mouth conditions, thus further studies need to be performed to provide insightful data regarding the cytotoxicity/cytocompatibility of these dental implants over a longer period of immersion or under various conditions caused by changes in saliva pH or other factors such as corrosion, mechanical stress, or bacterial colonization.

## 5. Conclusions

Within the current experimental conditions, different types of implants on short-term exposure set-up showed specific physical characteristics of the surface topography of the implants. Still, regarding the biosafety profile, the stainless steel implant developed a low cytotoxic effect that might have been non-detectable in clinical trials. However, the cytotoxic activity induced on HGF cells was not very significant and blood contact impairment was also reduced, but vigilance is required in Ni-sensitive patients. The other two orthodontic implants based on titanium metal did not manifest important cytotoxicity on HGF cell population and, further, on a simple contact blood test, the toxicological data show no toxicity. All these aspects have acquired the biosafety profile of the test orthodontic devices within the limitations of the present study and offered a reliable indicator for clinical applications.
